# From farm to plate: Spatio-temporal characterization revealed compositional changes and reduced retention of γ-oryzanol upon processing in rice

**DOI:** 10.3389/fnut.2022.1040362

**Published:** 2022-11-17

**Authors:** Swarnadip Ghosh, Haritha Bollinedi, S. Gopala Krishnan, Aditi Kundu, Anupama Singh, Prolay Kumar Bhowmick, Archana Singh, Mariappan Nagarajan, Kunnummal Kurungara Vinod, Ranjith Kumar Ellur, Ashok Kumar Singh

**Affiliations:** ^1^Division of Genetics, ICAR-Indian Agricultural Research Institute, New Delhi, India; ^2^Division of Agricultural Chemicals, ICAR-Indian Agricultural Research Institute, New Delhi, India; ^3^Division of Biochemistry, ICAR-Indian Agricultural Research Institute, New Delhi, India; ^4^ICAR-Indian Agricultural Research Institute, Rice Breeding and Genetics Research Centre, Aduthurai, India

**Keywords:** γ-oryzanol, antioxidants, cooking, grain development, rice bran oil, biofortification

## Abstract

**Background:**

Antioxidants detain the development and proliferation of various non-communicable diseases (NCDs). γ-oryzanol, a group of steryl ferulates and caffeates, is a major antioxidant present in rice grain with proven health benefits. The present study evaluated the distribution and dynamics of γ-oryzanol and its components in spatial and temporal scales and also delineated the effect of processing and cooking on its retention.

**Methods:**

Six rice varieties (four Basmati and two non-Basmati) belonging to *indica* group were analyzed at spatial scale in four different tissues (leaf blades, leaf sheaths, peduncle and spikelets) and temporal scale at three developmental stages (booting, milky and dough). Additionally, the matured grains were fractioned into husk, embryo, bran, and endosperm to assess differential accumulation in these tissues. Further, milling and cooking of the samples was done to assess the retention upon processing. After extraction of γ-oryzanol by solvent extraction method, individual components were identified by UPLC-QToF-ESI-MS and quantified by RP-HPLC.

**Results:**

The non-seed tissues were significantly different from the seed tissues for composition and quantitative variation of γ-oryzanol. Cycloartenyl caffeate was predominant in all the non-seed tissues during the three developmental stages while it showed significant reduction during the growth progression toward maturity and was totally absent in the matured grains. In contrary, the 24-methylenecycloartanyl ferulate, campesteryl ferulate and β-sitosteryl ferulate showed significant increment toward the growth progression to maturity. Milling caused significant reduction, retaining only an average of 58.77% γ-oryzanol. Cooking of brown rice in excess water showed relatively lower average retention (43.31%) to samples cooked in minimal water (54.42%). Cooked milled rice showed least mean retention of 21.66%.

**Conclusion:**

The results demonstrate prominent compositional variation of γ-oryzanol during different growth stages. For the first time, the study demonstrated that ferulate esters of γ-oryzanol were predominant in the seed tissues while caffeate esters were dominant in non-seed tissues. Basmati cultivars show differential expression of γ-oryzanol and its components compared to non-Basmati cultivars. Cooking in excess water causes maximum degradation of γ-oryzanol. Post-harvest losses due to milling and cooking indicate the necessity of biofortification for γ-oryzanol content in rice grain.

## Introduction

In the living organisms, free radicals are generated naturally as biproducts of endogenous metabolism. External factors like exposure to environmental pollutants, radiations, industrial solvents, medicines and pesticides further exaggerate the production of free radicals in the cell. The oxygen containing free radicals, also called reactive oxygen species (ROS) cause considerable damage to cell membranes and biomolecules like DNA, lipids and proteins leading to their degeneration. In humans, the oxidative stress causes several anomalies like cardiovascular disorders, cancer, ageing, auto-immune and neuro-degenerative diseases (1–4). Therefore, prevention of oxidative damage is the key to lower the risk of chronic and degenerative diseases. Antioxidants can mitigate the oxidative stress by neutralizing the ROS through donating free electrons. Thus, maintenance of balance between ROS and antioxidants is critical to address the adverse consequences of oxidative damage to the cell.

Rice is the major cereal crop sustaining the life and livelihood of more than half of the world population (5). It is grown in more than 100 countries and as many as 4,00,000 accessions of rice adapted to diverse soil and water regimes are being cultivated since ages (6, 7). Rice, in general, is mainly consumed as polished white rice (also called milled rice) to meet the calorific requirements. More recently, consumption of brown rice is gaining importance, as it retains major nutraceutical components like γ-oryzanol, tocopherols, tocotrienols, policosanols, octacosanol, squalene and γ-aminobutyric acid (GABA), having beneficial health effects (8, 9). γ-oryzanol is a predominant antioxidant in the rice grain. It is a family of ferulic acid and caffeic acid esters of phytosterols and triterpene alcohol (10). Although 25 different components were identified in rice, cycloartenyl ferulate, 24-methylenecycloartanyl ferulate, campesteryl ferulate, β-sitosteryl ferulate constitute a major portion of γ-oryzanol (11, 12). The health benefits of γ-oryzanol includes antioxidative, antidiabetic, anti-tumorous, anti-inflammatory, anti-cancerous and hypolipidemic action (13–18). As a result, there has been significant growth in the global γ-oryzanol market in recent years. Further, the increased utilization of γ-oryzanol in industrial products such as dietary and sports supplements, pharmaceuticals, cosmetics and animal feed has led to a significant surge in the market demand for γ-oryzanol during the last decade, with an estimated γ-oryzanol demand of 14.8 thousand tons in 2020, which is expected to increase to 20.6 thousand tons by 2027 (19).

In spite of its huge industrial and economic potential, there is very limited understanding on the composition of γ-oryzanol in rice grain and the physiological mechanisms that facilitate its spatial and temporal distribution in rice plant and partitioning into various grain compartments. Although tissue-specific, stage-specific and cultivar-specific accumulation of metabolites like flavonoids (20) and phenolamides (21) in rice have been previously reported, focused studies demonstrating the spatio-temporal dynamics of γ-oryzanol are lacking. Kim et al. (22) attempted to understand the stage specific expression of lipid substances including γ-oryzanol, tocols, fatty acids and policosanols (22), nevertheless, the study focused on the dynamics in panicle tissue *per se*, while the compositional analysis in vegetative tissues was disregarded.

In view of this, the current study was planned to characterize the spatial variation in accumulation of γ-oryzanol in various tissues namely leaf blade, leaf sheath, peduncle and spikelet at temporal scale through its assessment at different developmental stages. We aim to understand the compositional dynamics of γ-oryzanol in different tissues of rice plant and also elucidate if the biosynthetic pathways of γ-oryzanol in the vegetative tissues and reproductive tissues operate independently or interconnected to each other. Apart from these compositional dynamics, the partitioning of γ-oryzanol and its components within rice grain and the effect of post-harvest processing and cooking on its retention has also been investigated.

## Materials and methods

### Plant material

The study involved six rice varieties belonging to *indica* subgroup of the species *Oryza sativa* L., four of which were Basmati rice varieties namely, Pusa Basmati 1, Pusa Basmati 1121, Pusa Basmati 6 and Taraori Basmati and two were of non-Basmati rice varieties namely, Swarna and BPT 5204. These six *indica* rice varieties were selected since these are very popular and widely cultivated in India. The seeds of these varieties were sourced from the rice germplasm collection maintained in the Rice Genetics Lab, ICAR-Indian Agricultural Research Institute (ICAR-IARI), New Delhi.

### Chemicals

Chromatography grade organic solvents like methanol (purity 99.8%), acetonitrile (purity 99.9%), 2-propanol (purity 99.7%) and HPLC grade water acidified with 1% acetic acid were purchased from Merck Life Science Pvt Ltd., Mumbai, India, while the standard γ-oryzanol was purchased from Sigma-Aldrich Co., St. Louis, MO, USA.

### Field experimentation and sample collection

All the six varieties were grown in the experimental fields of ICAR-IARI, New Delhi, India (28.04*^o^*N 77.12*^o^*E, MSL-228m) during the Kharif or wet season (May–October), 2021 under hot and tropical humid climatic conditions. The soils in the experimental fields of ICAR-IARI, New Delhi are sandy loam type with neutral soil pH of 7.4–7.8. The germinated seeds were sown in raised, wet bed and further transplanting of 28-days old seedlings was done in puddled field with a row to row spacing of 20 cm and plant to plant spacing of 15 cm. The recommended dose of fertilizers, 120:60:60 kg ha^–1^ of N, P_2_O_5_ and K_2_O with 1/3rd of N, and full dose of P and K as basal and the remaining dose of N in two splits, one each at tillering and panicle initiation stages was applied. The fields were monitored regularly for any abiotic/biotic stresses and appropriate control measures were adapted.

The samples including leaf blade, leaf sheath, peduncle (referred to as non-seed tissues in the later sections) and spikelets (referred to as seed tissue in the later sections) were collected at three different developmental stages of rice growth, *viz*., booting, milky and dough stage. As the varieties involved in the study varied for their days to 50% flowering and maturity, fields were monitored regularly and the samples were collected at appropriate stages, separately for each of these varieties. Samples were collected from three biological replicates which were further pooled to generate two technical replicates for the analysis of γ-oryzanol. The samples were flash frozen in liquid N_2_ immediately after separation from the plant and then stored at -80°C, until analysis of γ-oryzanol. Grains were harvested at physiological maturity, dried to safe moisture level and stored until analysis. To characterize the partitioning of γ-oryzanol in grains, the grains were fractioned into husk, embryo, bran and endosperm. The embryos from the brown rice were carefully separated using forceps and the non-embryonic brown rice was further subjected to milling to collect the bran samples constituting seed coat, pericarp and aleurone layers.

### Milling and cooking of rice samples

To assess the effect of post-harvest processes like milling and cooking about 1 Kg of paddy sample of the six varieties were dehulled using Satake THU35B thresher (Satake, Japan) to obtain brown rice. A portion of brown rice was further processed to obtain milled rice by removing the bran layers using a rice polisher (Mini Lab Rice Polisher Model K-710, Krishi International, Hyderabad, India). To emulate the most common methods of cooking rice in majority of the households, two different methods were employed for cooking the brown rice samples, one using minimal water i.e., two portion of water with one portion of rice (2:1) by volume and the other with excess water (5:1, five portion of water with one portion of rice by volume). Prior to the cooking in rice cookers (Bajaj Majesty RCX 1 Mini 0.4-Liter), the rice samples were kept soaked for 1 h after adding 2 and 5 volumes of water according to the treatment. The samples of excess water treatment (5:1) were allowed to cook until the rice grains swelled to 3/4th of their volume, then the excess water was drained through the hole in the lid and the samples were further cooked until the cook indicator of the rice cooker turns to warm mode. The samples of the 2:1 treatment were cooked without any interruption until the indicator turns to warm mode. The milled rice samples were cooked only with 2:1 water treatment.

### Extraction of γ-oryzanol

The samples were treated with liquid N_2_ and crushed by using Ez-lyzer tissue homogenizer (Genetix, India). γ-oryzanol was extracted through the solvent extraction protocol as presented in Bollinedi et al. (23). Briefly, 25 mg of homogenized samples for plant tissues and 50 mg of grain samples and cooked rice samples were weighed into test tubes and 1 ml HPLC grade methanol was added. The samples were vortex mixed for about a 1 min and then incubated in water bath at 60°C for 1 h. Subsequently, samples were centrifuged at 825×*g* for 10 min. The supernatant after centrifugation was collected in clean tubes and the residue was further extracted twice with methanol. The pooled supernatants were further dried under a stream of N_2_ (99.999% pure) and finally the samples were redissolved in 1 ml of methanol, filtered through a syringe filter having pore size of 0.22 μ for HPLC analysis.

### UPLC-QToF-MS/MS based identification of individual components of γ-oryzanol

Samples were characterized to identify individual component of γ-oryzanol using liquid chromatography and mass spectrometric analysis in a UPLC-QToF-ESI-MS-MS (Waters^®^, UK). The samples (5 μg/ml, each) were prepared in LCMS grade acetonitrile and separated through a stationary phase, UPLC BEH C_18_ (2.1 × 100 mm, 1.8 μm) using a gradient system of acetonitrile with 0.1% formic acid in channel A and water with 0.1% formic acid in channel B. The volume of injection was 5 μl for all the samples. Run time of each sample was 75 min. The mass spectrometer conditions were tuned and maintained as capillary voltage of 2.97 kV, sampling cone 30 V, extraction cone 4.99 V, source temperature 150°C, desolvation temperature 300°C, mass range 50–1,000. For mass spectrometer calibration, 0.5 mM sodium formate was used. The lock spray, the reference mass leucine enkephalin (m/z 556.2771 in positive and m/z 554.2670 in negative polarity) was used for mass correction with a flow rate 10 μl/min at the concentration of 1 μg/ml at every 15 s interval. Data analysis was done in MassLynx version 5.1. Individual compound was identified based on their accurate mass and corresponding mass fragmentation pattern.

### Separation of components of γ-oryzanol and quantification in reversed phase high performance liquid chromatography

Individual components of γ-oryzanol were separated and quantified following a protocol given by Chen and Bergman (24) with some customs (24) in a reversed phase high performance liquid chromatography (RP-HPLC) system of Waters (Waters Pvt. Ltd.) equipped with Waters 600 controller, a 717 plus autosampler and a 2998 photodiode array (PDA) detector. The compound separation was carried on a XBridge™ C18 chromatography column (4.6 × 250 mm, i.d. 5 μm). The mobile phase was set using four different HPLC grade solvents, including acidified water with 1% aqueous acetic acid (v/v) (A), methanol (B), acetonitrile (C) and 2-propanol (D). The flow rate was set as 2 ml/min (constant). Gradient mode of the solvent system with 5% A, 45% B, 45% C and 5% D by volume in the initial 8 min followed by 70% B, 25% C and 5% D for the next 6 min was used. Detection of γ-oryzanol was done using photodiode array (PDA) detector at the wavelength of 325 nm.

### Statistical analysis

The peak areas of the individual components of γ-oryzanol were processed to percentage proportion of total γ-oryzanol. Descriptive statistics including mean, standard deviation (SD) and standard error (SE) were worked out in MS-Excel version 2019. Fold change differences were calculated among the samples and conditions and the significance of fold change was determined using Tukey’s multiple comparison test (25). The figures were prepared using MS-Excel version 2019 and R software version 4.2.1.

## Results

### Identification of components of γ-oryzanol

Different derivatives of γ-oryzanol were characterized in the samples using UPLC-QToF-ESI-MS, operated in negative mode. Components were tentatively identified from accurate mass along with their corresponding mass fragmentation pattens. Mass spectral data of nine major peaks revealed mass similarities with γ-oryzanol components viz., 24-methylenecycloartanyl-p-coumarate, Δ7-stigmastenyl ferulate, stigmasteryl ferulate, cycloartenyl caffeate, cycloartenyl ferulate, campesteryl caffeate, 24-methylenecycloartanyl ferulate, campesteryl ferulate and β-sitosteryl ferulate from exact mass of 585.4296, 587.4061, 587.4077, 587.4091, 601.4246, 561.3996, 615.4399, 575.4063 and 589.4232, respectively.

Mass fragmentation pattern of the most abundant molecular ion [M]^–^ peak of cycloartenyl ferulate at m/z 601.4246 showed major daughter ion peaks at m/z 425, originated due to ester cleavage and subsequent removal of acidic moiety. Apart from these major peaks, other minor components such as isomers of cycloartenyl ferulate (m/z 601.4246), campesteryl cinnamate (m/z 529.4078), campestanyl ferulate (m/z 577.4286), stigmastanyl ferulate (m/z 591.4457), 24-methylenecycloartenyl ferulate (m/z 613.4195), campestanyl caffeate (m/z 563.4076), cycloartenyl cinnamate (m/z 555.4210), 24-methylenecholesteryl ferulate (m/z 573.3914), cycloartanyl ferulate (m/z 603.4452), hydroxydehydrocycloartenyl ferulate (m/z 615.4085) and hydroxycycloartenyl ferulate (m/z 617.4146) were also being detected (Table 1). Presence of these steroidal components of γ-oryzanol were also confirmed from their accurate mass spectral data including major daughter ion peaks.

**TABLE 1:** Tentative composition of γ-oryzanol obtained from the extracts of plant samples in rice across the developmental stages.

S. No	Tentative compound	Formula	Neutral mass (Da)	Observed mass (Da)	Adduct	Error mass (δ)
**Major compounds**						
1	24-methylenecycloartanyl-*p*-coumarate	C_40_H_58_O_3_	586.4388	585.4296	H^–^	–2.40
2	Δ7-stigmastenyl ferulate	C_39_H_56_O_4_	588.4181	587.4061	H^–^	–7.09
3	Stigmasteryl ferulate	C_39_H_56_O_4_	588.4181	587.4077	H^–^	–4.37
4	Cycloartenyl caffeate	C_39_H_56_O_4_	588.4181	587.4091	H^–^	–1.99
5	Cycloartenyl ferulate	C_40_H_58_O_4_	602.4337	601.4246	H^–^	–2.11
6	Campesteryl caffeate	C_37_H_54_O_4_	562.4024	561.3996	H^–^	8.94
7	24-methylenecycloartanyl ferulate	C_41_H_60_O_4_	616.4494	615.4399	H^–^	–2.71
8	Campesteryl ferulate	C_38_H_56_O_4_	576.4181	575.4063	H^–^	–6.89
9	β-sitosteryl ferulate	C_39_H_58_O_4_	590.4337	589.4232	H^–^	–4.52
**Minor compounds**						
1	Isomers of cycloartenyl ferulate	C_40_H_58_O_4_	602.4337	601.4246	H^–^	–2.11
2	Campesteryl cinnamate	C_37_H_54_O_2_	530.4126	529.4078	H^–^	5.71
3	Campestanyl ferulate	C_38_H_58_O_4_	578.4337	577.4286	H^–^	4.72
4	Stigmastanyl ferulate	C_39_H_60_O_4_	592.4494	591.4457	H^–^	6.97
5	24-methylenecycloartenyl ferulate	C_41_H_58_O_4_	614.4337	613.4195	H^–^	–10.37
6	Campestanyl caffeate	C_37_H_56_O_4_	564.4181	563.4076	H^–^	–4.73
7	Cycloartenyl cinnamate	C_39_H_56_O_2_	556.4283	555.4210	H^–^	0.95
8	24-methylenecholesteryl ferulate	C_38_H_54_O_4_	574.4024	573.3914	H^–^	–5.52
9	Cycloartanyl ferulate	C_40_H_60_O_4_	604.4494	603.4452	H^–^	5.96
10	Hydroxydehydrocycloartenyl ferulate	C_40_H_56_O_5_	616.4129	615.4085	H^–^	5.56
11	Hydroxycycloartenyl ferulate	C_40_H_58_O_5_	618.4286	617.4146	H^–^	–9.98

**FIGURE 1:**
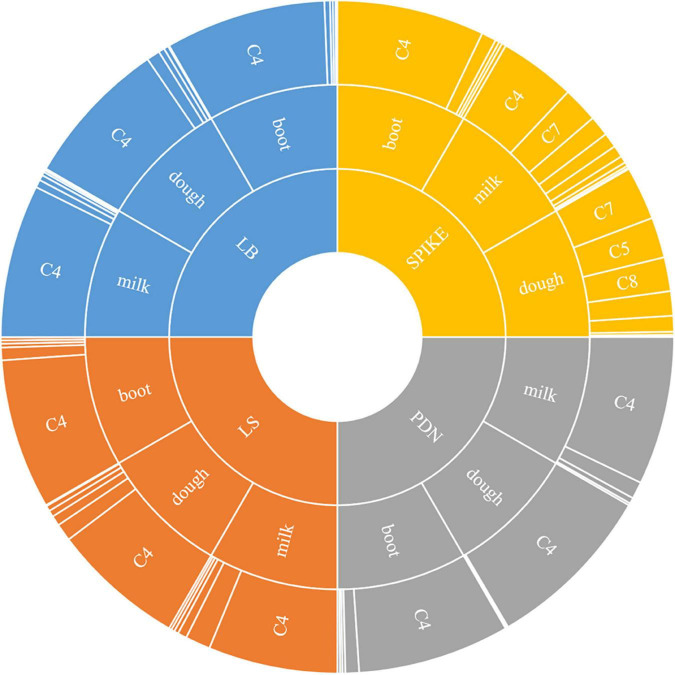
Sunburst chart showing spatio-temporal changes of various components of γ-oryzanol in four different tissues (LB: leaf blades; LS: leaf sheaths; PDN: peduncle; Spike: spikelets) across three different developmental stages (Boot, Milk, and Dough). The size of the segment reflects the percentage proportion of a particular component to total γ-oryzanol. C1: 24-methylenecycloartanyl-*p*-coumarate; C2: Δ7-stigmastenyl ferulate; C3: stigmasteryl ferulate; C4: cycloartenyl caffeate; C5: cycloartenyl ferulate; C6: campesteryl caffeate; C7: 24-methylenecycloartanyl ferulate; C8: campesteryl ferulate; C9: β-sitosteryl ferulate.

The stage-specific, tissue-specific and cultivar-specific expressions of these above components showed that there was significant temporal, spatial and compositional variation in rice plant as detailed below (Figure 1).

### Temporal variation of components of γ-oryzanol at different developmental stages in rice

The seed tissue i.e., spikelets behaved differently with respect to both composition and the quantitative variation for the individual components of γ-oryzanol when compared to the non-seed tissues *viz*. leaf blades, leaf sheath, and peduncle during the three developmental stages analyzed (Figure 1). For instance, the 24-methylenecycloartanyl-*p*-coumarate, Δ7-stigmastenyl ferulate, stigmasteryl ferulate and cycloartenyl caffeate showed a decreasing trend from booting stage to milky stage and further to dough stage in the spikelet tissue, while these compounds showed an increasing trend from booting to milky stage and then decreased from milky to dough stage in other vegetative parts. Cycloartenyl caffeate showed almost 2-fold reduction from booting stage to milky stage, while from milky to dough stage, there was about 3.13-fold reduction. Cycloartenyl ferulate content showed non-significant differences in the spikelet tissue at the booting stage, while it increased 6.52-fold from booting stage to milky stage and further 2-fold increase from milky stage to dough stage reaching to a level that accounts almost an average of 23.33% of total γ-oryzanol in the spikelet tissues at dough stage.

In the non-seed tissues, cycloartenyl ferulate remained to be the minor component during all the three developmental stages. The spikelet tissue specific components 24-methylenecycloartanyl ferulate, campesteryl ferulate and β-sitosteryl ferulate showed progressive increments in accumulation from booting stage to dough stage. Maximum spike in these components in spikelet was recorded during the developmental progression from booting stage to milky stage as compared to that of milky to dough stage progression. For instance, the compound 24-methylenecycloartanyl ferulate showed 21.5-fold increase from booting stage to milky stage and a further 1.51-fold increase from milky to dough stage, while campesteryl ferulate showed about 15.66 and 2.29-fold increase from booting to milky and milky to dough stages, respectively. The compound β-sitosteryl ferulate was totally absent in the booting stage, while it accounted to almost 4.03 and 9.1% of total γ-oryzanol in milky and dough stages, respectively. Overall, in the spikelet tissues, the order of abundance of the components in terms of their proportional contribution to total γ-oryzanol in the booting stage was cycloartenyl caffeate > Δ7-stigmastenyl ferulate > 24-methylenecycloartanyl-*p*-coumarate > cycloartenyl ferulate > 24-methylenecycloartanyl ferulate > campesteryl caffeate > campesteryl ferulate > β-sitosteryl ferulate = stigmasteryl ferulate (Supplementary Table 1).

**FIGURE 2:**
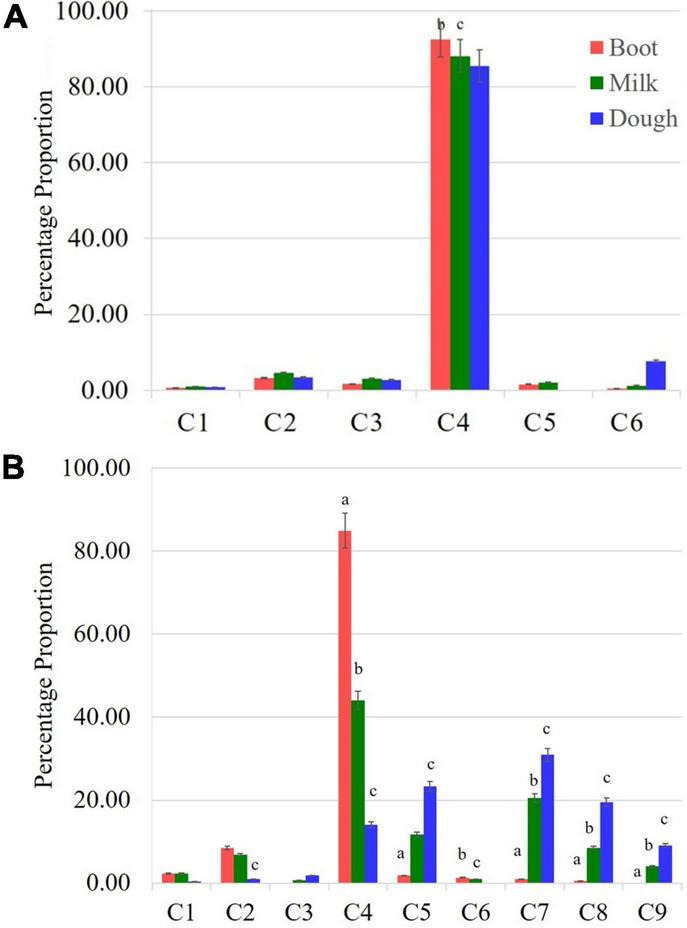
Compositional variation for components of γ-oryzanol between non-seed (leaf blades **(A)** and seed tissue [Spikelets **(B)**]. Leaf blades have only six components while nine components were found in spikelet tissue. C1: 24-methylenecycloartanyl-*p*- coumarate; C2: Δ7-stigmastenyl ferulate; C3: stigmasteryl ferulate; C4: cycloartenyl caffeate; C5: cycloartenyl ferulate; C6: campesteryl caffeate; C7: 24-methylenecycloartanyl ferulate; C8: campesteryl ferulate; C9: β-sitosteryl ferulate; significant difference between boot and milk stages **(a)**, milk and dough stages **(b)**, boot and dough stages **(c)**.

A significant change in the composition of total γ-oryzanol was evident during the developmental progression from booting to dough stage. The order of abundance of the components at the dough stage was 24-methylenecycloartanyl ferulate > cycloartenyl ferulate > campesteryl ferulate > cycloartenyl caffeate > β-sitosteryl ferulate > stigmasteryl ferulate > Δ7-stigmastenyl ferulate > 24-methylenecycloartanyl-*p*-coumarate. There was a clear reversal in trends of the components between pre- and post-anthesis stages as the components which showed higher expression in the pre-anthesis stage i.e., booting stage turned out to be the minor components in the post anthesis stage i.e., dough stage (Figure 2A). It was observed that these components, viz., 24-methylenecycloartanyl-*p*-coumarate, Δ7-stigmastenyl ferulate and campesteryl caffeate further decreased toward late maturity (Figure 2B) and they were totally absent in the matured grains. In the non-seed tissues, cycloartenyl caffeate followed by Δ7-stigmastenyl ferulate remained to be the major components of total γ-oryzanol in all the developmental stages (Supplementary Tables 2–4). This showed that the developmental stage has a significant impact on the composition of γ-oryzanol in the seed tissue, while its influence on the composition in non-seed tissues was minimal.

### Spatial variation of components of γ-oryzanol in different tissues of rice

**FIGURE 3:**
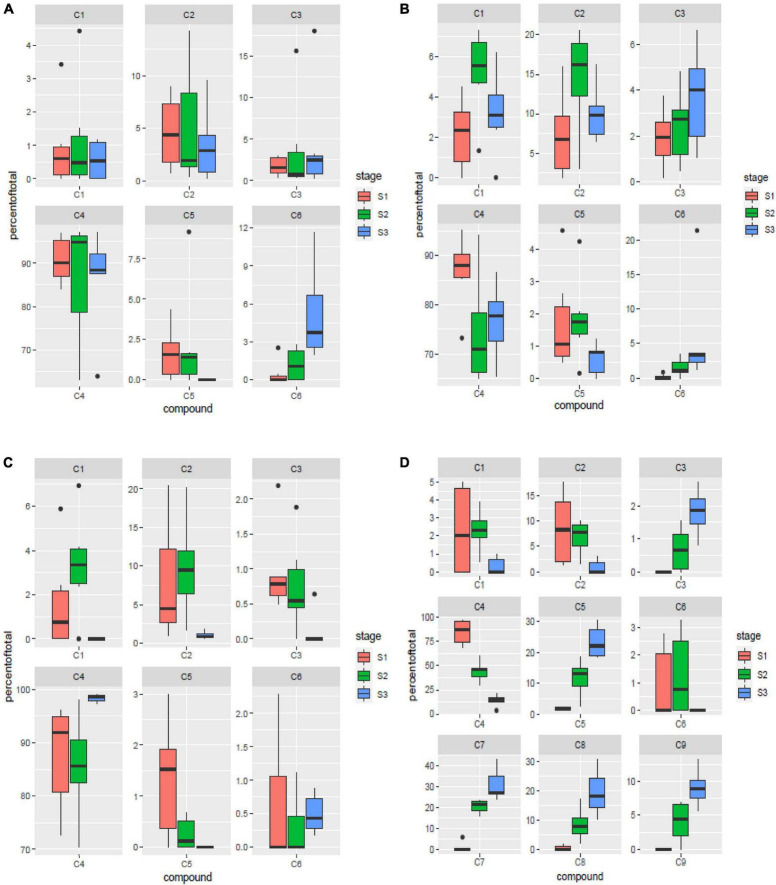
Spatial variation for components of γ-oryzanol in leaf blades **(A)**, leaf sheaths **(B)**, peduncles **(C)** and spikelets **(D)**. S1: booting stage; S2: milky stage and S3: dough stage. C1: 24-methylenecycloartanyl-*p*-coumarate; C2: Δ7-stigmastenyl ferulate; C3: stigmasteryl ferulate; C4: cycloartenyl caffeate; C5: cycloartenyl ferulate; C6: campesteryl caffeate; C7: 24- methylenecycloartanyl ferulate; C8: campesteryl ferulate; C9: β-sitosteryl ferulate.

Six of the nine major components identified in the study *viz*. 24-methylenecycloartanyl-*p*-coumarate, Δ7-stigmastenyl ferulate, stigmasteryl ferulate, cycloartenyl caffeate, cycloartenyl ferulate and campesteryl caffeate were present in all the four tissues analyzed. Of these six components, 24-methylenecycloartanyl-*p*-coumarate and Δ7-stigmastenyl ferulate showed relative abundance of expression in the leaf sheath tissues followed by peduncle and spikelet tissues, while least expression of these compounds was observed in the leaf blades. Stigmasteryl ferulate was observed to show higher expression in leaf sheaths and leaf blades, while least quantity was observed in peduncles. Cycloartenyl caffeate is the major compound constituting almost 90.5% of total γ-oryzanol content in the peduncle tissues, followed by 88.73% in leaf blades, 79.26% in leaf sheaths, and 47.71% in spikelet tissues across the three developmental stages. The significantly higher expression of cycloartenyl ferulate was recorded in spikelets, while all other tissues showed very less accumulation. Campesteryl caffeate showed highest expression in leaf blades followed by leaf sheaths, while its expression was lowest in peduncle and spikelet tissues (Figures 3A–D). The remaining three components *viz*. 24-methylenecycloartanyl ferulate, campesteryl ferulate and β-sitosteryl ferulate were very specifically observed in spikelet tissue. Of these three components, 24-methylenecycloartanyl ferulate was most abundant (17.44%) followed by campesteryl ferulate (9.54%) and β-sitosteryl ferulate (4.38%) (Figure 3D). The specific occurrence of 24-methylenecycloartanyl ferulate, campesteryl ferulate and β-sitosteryl ferulate in the spikelet tissues indicate that their biosynthesis is triggered independently in the spikelets. The translocation of these compounds from the vegetative tissues including leaf blade and leaf sheath does not contribute to their content in the spikelets.

### Spatio-temporal variation for total γ-oryzanol content

In the present study, the change in total γ-oryzanol content was analyzed not only in the seed tissues but also in the non-seed (vegetative) tissues. The total γ-oryzanol content followed a decreasing trend from booting stage to dough stage in all the three non-seed tissues. In the leaf blades, there was a reduction of about 13.35% from booting to milky stage and from milky to dough stage a maximum reduction of 36.81%. Similar trend was observed in leaf sheath tissues with about 8.11 and 29.95% reduction from booting to milky and milky to dough stages, respectively. Though peduncle tissues also showed a decreasing trend with progression of growth stage, a greater reduction was observed from booting to milky (27.56%) as compared to that of milky to dough stage (23.89%). The seed tissue i.e., spikelets showed a trend different from that of non-seed tissues. The total γ-oryzanol content was found to reduce from booting to milky stage (33.90%), while it increased by 16.32% from milky to dough stage.

### Cultivar-specific variation for γ-oryzanol and its components in matured grains

**FIGURE 4:**
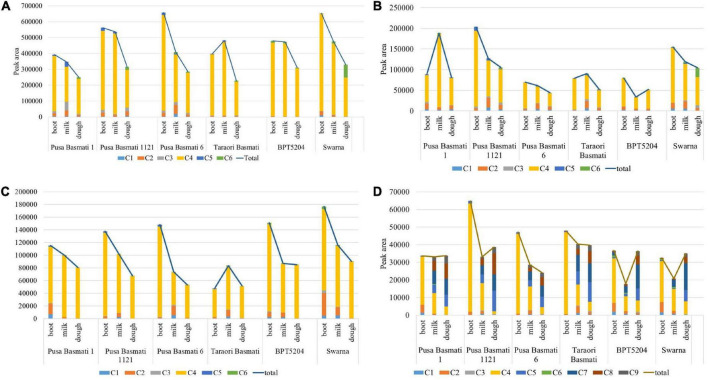
Cultivar-specific variation for total γ-oryzanol and its components in **(A)** leaf blade, **(B)** leaf sheath, **(C)** peduncle and **(D)** spikelet; C1: 24-methylenecycloartanyl-*p*-coumarate; C2: Δ7-stigmastenyl ferulate; C3: stigmasteryl ferulate; C4: cycloartenyl caffeate; C5: cycloartenyl ferulate; C6: campesteryl caffeate; C7: 24- methylenecycloartanyl ferulate; C8: campesteryl ferulate; C9: β-sitosteryl ferulate.

A significant variation for total γ-oryzanol and its components was observed among the varieties analyzed in the study. In leaf blades during the booting stage, the Basmati cultivar, Pusa Basmati 1121 and non-Basmati cultivar, Swarna showed relatively higher concentration of total γ-oryzanol, while it was low in Pusa Basmati 1 (Figure 4A). Though all the varieties depicted a decreasing trend for total γ-oryzanol content during the progression from booting to dough stage, maximum reduction in quantity was observed in Pusa Basmati 6 (56.57%) in leaf blades. The composition of γ-oryzanol in Basmati varieties was found to be significantly different from non-Basmati varieties. For example, all the four Basmati varieties analyzed showed complete absence of campesteryl caffeate during booting and milky stages, while its presence was still observed in non-Basmati varieties in the leaf blade and peduncle tissues. Further, in these tissues, cycloartenyl ferulate was absent in the non-Basmati varieties in all the three growth stages, while it was present in Basmati varieties during booting and milky stages. In the leaf sheaths and peduncles, campesteryl caffeate was found to be absent in the Basmati varieties (with the exception of Pusa Basmati 1 and Taraori Basmati that showed minute quantity in the milky stage) while it was present in the non-Basmati varieties BPT 5204 and Swarna (Figures 4B–C). However, it showed an increasing trend in the leaf sheaths, while opposite was observed in the peduncle tissues. In the spikelet tissues, Pusa Basmati 1121 showed relatively higher concentration of total γ-oryzanol across all the stages followed by Taraori Basmati (Figure 4D). Significant differences were also observed between Basmati and non-Basmati varieties for the components of γ-oryzanol in the spikelet tissues. The Basmati accessions showed the accumulation of stigmasteryl ferulate in milky and dough stages, while its expression was delayed to dough stage in the non-Basmati accessions.

### Compartmentalization of γ-oryzanol in different tissues of rice grain

In order to understand the differential accumulation of γ-oryzanol in different sub-sections of the rice grain, the γ-oryzanol content was estimated in husk, bran, embryo and endosperm in the matured grains. The bran layers showed highest accumulation of γ-oryzanol ranging from 107.53 mg/100 g (Pusa Basmati 6) to 129.67 mg/100 g (Pusa Basmati 1121). It was followed by the embryo samples in terms of abundance of γ-oryzanol, which was in the range of 46.97 mg/100 g (Swarna) to 59.29 mg/100 g (BPT 5204). The quantity of total γ-oryzanol in the outer husks of rice grain was found to be ranging from 5.23 (Pusa Basmati 1121) to 37.79 (Pusa basmati 1) mg/100 g. The starchy endosperm which is the most widely consumed tissue in rice showed the lowest γ-oryzanol content ranging from 11.44 (Pusa Basmati 1) to 14.33 (Taraori Basmati) mg/100 g. In all these tissues, 24-methylenecycloartanyl ferulate was found to be the predominant component, whereas stigmasteryl ferulate and cycloartenyl caffeate constituted a lower proportion of total γ-oryzanol (Supplementary Table 5).

### Effect of milling on retention of γ-oryzanol and its components

Rice is popularly consumed as milled/white rice obtained after the processing steps including de-husking (removal of outer husks) and milling/polishing (removal of outer bran layers constituting aleurone and embryo). Hence, the retention of γ-oryzanol after these processing steps has been investigated in the present study. Here, we observed significant retention of γ-oryzanol of 41.23% upon milling (Table 2). There was considerable variation for the percentage retention of total γ-oryzanol content in the varieties, due to milling. For instance, the non-Basmati cultivar, BPT 5204 showed the maximum reduction of 68.42%, retaining a minimum of 31.58% while maximum retention of 49.64% was noticed in the Basmati cultivar, Pusa Basmati 1121 (Supplementary Figure 1). The individual components of γ-oryzanol also showed marked variation for the percentage retention. On an average, cycloartenyl caffeate and β-sitosteryl ferulate recorded a retention of 31.90 and 38.34%, respectively. The major components *viz*. 24-methylenecycloartanyl ferulate, cycloartenyl ferulate and campesteryl ferulate showed about 39.58, 42.79, and 46.62% retention, respectively.

**TABLE 2:** Summary of retention of γ-oryzanol and its components upon processing and milling.

Variety	Concentration (mg/100 g)					% Retention			
		
	BR	MR	Cooked BR (T1)	Cooked BR (T2)	Cooked MR	Milling	Cooking of BR (T1)	Cooking of BR (T2)	Cooking of milled rice*
**Basmati**									
Pusa Basmati 1	28.80	11.44	15.64	13.90	6.28	39.71	54.30	48.25	21.81
Pusa Basmati 1121	25.26	12.54	15.14	12.57	6.59	49.65	59.96	49.77	26.09
Pusa Basmati 6	27.51	12.89	18.60	14.33	6.75	46.86	67.61	52.08	24.52
Taraori Basmati	33.33	14.33	21.18	16.04	6.71	43.00	63.55	48.14	20.14
**Non-Basmati**									
BPT 5204	45.23	14.28	17.67	15.03	6.63	31.57	39.06	33.23	14.65
Swarna	33.93	12.42	14.26	9.63	7.71	36.61	42.02	28.38	22.73
Mean ± SE	32.34 ± 2.92	12.98^a^ ± 0.46	17.08^b^ ± 1.05	13.58^c^ ± 0.92	6.78^d^ ± 0.20	41.23	54.42	43.31	21.66
SD	7.15	1.13	2.58	2.26	0.49				

BR, brown rice; MR, milled rice; *% retention calculated with respect to the amount present in brown rice samples. T1, brown rice cooked in minimal water (1: 2 proportion of grains and water, v/v); T2, brown rice cooked in excess amount of water (1: 5 proportion of grains and water, v/v). ^a,b,c,d^Indicate significant difference of means of MR, cooked BR (T1), cooked BR (T2) and cooked MR from BR.

### Effect of cooking on retention of γ-oryzanol and its components

Traditionally rice is cooked in large volume of boiling water and the excess water is drained off once rice is cooked. Under this method of cooking, the average percentage retention of total γ-oryzanol was observed to be 43.31% with a range of 28.38% (Swarna) to 52.08% (Pusa Basmati 6) (Table 2). However, cooking rice with minimal water (2:1 proportion), retained significantly higher percentage of mean total γ-oryzanol (54.42%) content (Supplementary Figure 1). Under minimal water cooking, the major components of γ-oryzanol viz. 24-methylenecycloartanyl ferulate, cycloartenyl ferulate and campesteryl ferulate also showed a higher retention percentage of 49.42, 59.07, and 57.99%, respectively. The milled rice cooked with minimal water showed only a minimum of 21.66% of total γ-oryzanol content. Among the components, campesteryl ferulate was retained to a greater extent of 25.04% followed by 24-methylenecycloartanyl ferulate (21.73%), cycloartenyl ferulate (20.82%) in cooked milled rice with minimal water.

## Discussion

### Compositional and quantitative changes in γ-oryzanol

Understanding the spatial and temporal distribution of bioactive compounds is essential, as it paves the way to identify the stage specific and tissue specific pattern of gene expression that ultimately drives the biosynthesis and accumulation of these compounds. Several key genes controlling various traits show specific expression during certain developmental stages and in certain specific tissues which is further regulated, modulated and fine-tuned by both the external and internal factors. A growing number of evidences in favor of spatio-temporal accumulation of various bioactive compounds like tocopherols, folic acid, mineral nutrients like Fe and Zn are being published (12, 26, 27).

The natural genetic variations for γ-oryzanol in grains of different rice germplasms were explored previously by several studies (11, 12, 28, 29). Nevertheless, there are scanty evidences on the spatial and temporal pattern of γ-oryzanol accumulation in rice. In the present study, both qualitative and quantitative variation for stage specific and tissue specific accumulation of γ-oryzanol as well as its components have been recorded. Caffeate esters were found to be predominant during all the developmental stages in the vegetative (non-seed) tissues while they showed a decreasing trend from booting toward maturity stage in the spikelet tissue and were totally absent in the matured seed. Caffeate was reported to be the precursors of ferulate in grass species (30) and the increase in the proportion of ferulates toward maturity indicates a trigger in the expression of the genes responsible for the conversion of caffeate to ferulate during post-anthesis period in the reproductive tissues while these genes remain silent in the vegetative tissues showing predominance of caffeates throughout the development. This observation is novel to the study and would have implications in understanding the nutritional value of paddy straw as cattle feed and rice grain as food for human consumption as different components of γ-oryzanol exhibit different antioxidant activity (15).

During the developmental progression from booting to dough stage, an increase in cycloartenyl ferulate content was noticed in the spikelets of all the varieties under our study. Kim et al. also reported a significant increase in the proportion of cycloartenyl ferulate during the development of rice grains (22). However, they reported the increase to be variety-specific as the variety *Ilpum* exhibited a significant increase, while the variety *Dasan* reported no change in the proportion. Similarly, 24-methylenecycloartanyl ferulate and campesteryl ferulate exhibited increase in accumulation from booting to milky stage and milky to dough stage in our study. This finding is in contradiction with the report of Kim et al. who showed that there is no significant change in the relative proportion of 24-methylenecycloartanyl ferulate and campesteryl ferulate during the development of rice grain (22). This also indicates differential expression of γ-oryzanol in *indica* and *japonica* accessions as the cultivars analyzed by Kim et al. (22) belonged to the *japonica* group. Further, the present study also demonstrated differential accumulation of various components of γ-oryzanol in the Basmati cultivars belonging to aromatic sub-group of rice in comparison to non-Basmati cultivars. These evidences indicate sub-group-specific dynamics of γ-oryzanol expression. The study also demonstrated reduction of total γ-oryzanol from booting to milky stage (33.90%), although an increase to the extent of 16.32% was observed from milky to dough stage. There are additional reports available indicating the increased γ-oryzanol content in the mature seeds compared to the immature seeds (11, 31). This suggests that γ-oryzanol biosynthesis in rice is triggered during post-anthesis period and the peak production and accumulation in grains happens during the milky and dough stages concomitant to the starch accumulation in the developing grains.

The changes in concentration of γ-oryzanol were also accompanied with significant changes in its composition. Phytosterols, the precursors of γ-oryzanol, are synthesized in plants from squalene through a series of enzymatic reactions catalyzed by squalene-2,3-epoxidase and cycloartenol synthetase (CAS) enzymes to produce cycloartenol, the first committed triterpene alcohol of sterol biosynthesis pathway (32, 33). Subsequently alkylation of cycloartenol is catalyzed by two S-adenosyl-L-methionine-dependent methyltransferase (SMT) family enzymes, SMT1 and SMT2 (34). The relative expression of these genes modulates the levels of 24-methylenecycloartanol, campesterol, stigmasterol and sitosterol and their corresponding ferulate esters in the rice grain. Nevertheless, it is also essential to quantify the free sterols in rice grain in order to gain better understanding on the dynamics of ferulate ester synthesis.

### Effect of milling and cooking on retention of γ-oryzanol

In rice grain, bran and embryo tissues predominantly carry fat molecules that constitute the rice bran oil (RBO), wherein γ-oryzanol is present approximately at 2% level. (35, 36). This explains the differential accumulation pattern of γ-oryzanol in different grain tissues established by previous studies (12, 37) and the current study is yet another in line confirming the fact. Rice grains are normally processed before consumption, by which the lipid-rich bran layer and the germ are removed on milling. Processing therefore removes significant proportion of lipid soluble components including γ-oryzanols, tocopherols and β-carotene (23). Processed rice grains are nutritionally altered with changes in several nutrients, proteins, vitamin B and E, folic acid and minerals like Fe and Zn (8, 38, 39). The present study also documents a significant reduction of γ-oryzanol up to 58.77% upon milling. However, we observed a considerable variation for the percentage loss of γ-oryzanol upon milling among the varieties analyzed, which provides scope for selection of genotypes with higher retention upon milling.

As rice is cooked before consumption, it is also essential to understand the effect of cooking on the nutritional profile. Rice in households is cooked in excess water, often going up to 5 times the volume of rice grains. Upon cooking, the excess water is drained to keep the cooked grains friable and to prevent from lumping. The draining also helps in reducing the anti-nutritional factors like heavy metals such as As, Pb and Cd (40). Another common method is pressure cooking, which uses minimal water usually 2 times the volume of rice grains and does not involve draining. In this study, we have used both these treatments to see the effect of cooking on the γ-oryzanol content. Although cooking improves the process of digestion and absorption of nutrients, it leads to the reduction of vitamins like thiamine, niacin, folic acid and other B vitamins and Fe (40), β-carotene and vitamin A (41), bioactive compounds like GABA (42), vitamin E and minerals such as Zn and Ca (43, 44). Further, the method of cooking has significant influence on the retention of the nutrients. For instance, steaming is superior to other methods like sautéing, stir frying, roasting or baking as it causes comparatively lesser reduction of nutrients (45–48). Unlike other nutrients, studies were scanty on the retention of γ-oryzanol upon cooking. In the present study, we observed a significant loss of γ-oryzanol upon cooking in brown rice. Further, cooking in excess water (5:1) resulted in greater losses of γ-oryzanol in comparison to cooking in minimal (2:1) water. The effect of cooking heat on γ-oryzanol content remains unestablished, although a few studies show the effect of heat on the stability of γ-oryzanol during the extraction of RBO (49–51). Since the temperature remained the same for both the cooking treatments in this study, the excess amount of γ-oryzanol lost in the 5:1 treatment could be attributed to the leaching loss along with the drained water. The loss of γ-oryzanol in excess water is a novel observation of the present study. It would be interesting to know if the loss of γ-oryzanol in excess water is also associated with the loss of other lipids. A systematic analysis and quantitative estimation of the degradation of nutrients due to various processing methods provides insights to the plant breeders and enable them in determining the targets for various nutrients in the biofortification programme.

## Conclusion

With the increasing awareness on the benefits of healthy diet and proper nutrition, consumption of whole grain brown rice is gaining importance. γ-oryzanol is one of the major antioxidant compounds in rice grain with several nutraceutical properties. This study for the first time establishes the stage and tissue-specific profiling of γ-oryzanol and its constituents in the developing rice grains. We could also observe differential accumulation of components of γ-oryzanol in Basmati and non-Basmati accessions indicating sub-group specific differences. Furthermore, the grain processing and cooking methods have been found to influence the ultimate level of γ-oryzanol in the consumed rice. The study emphasizes the need of establishing the threshold limits of γ-oryzanol in rice grain to be achieved by the biofortification programme as well as to set the limits for post-harvest processing losses due to milling and cooking. Overall, this study throws light on the qualitative and quantitative variation for γ-oryzanol and its components as it reaches the plate of the consumer. The genotypic variability of γ-oryzanol constitution further calls for the investigations on the pathway genes, their tissue-specific and stage-specific expression and improvement by selection, introgression and gene editing in order to enhance the accumulation of γ-oryzanol in the rice grains.

## Data availability statement

The original contributions presented in the study are included in the article/Supplementary material, further inquiries can be directed to the corresponding author.

## Author contributions

SGh, HB, AK, ArS, AnS, PB, MN, and RE: investigation. HB and AKS: conceptualization, funding acquisition, and project administration. SGh, HB, KV, and SGo: methodology, validation, data curation, formal analysis, and drafting. HB, SGo, and KV: writing—review and editing. All authors have read and approved the final version of the manuscript.

## Supplementary material

Supplementary Figure 1 Radar plot depicting the percentage retention of γ-oryzanol upon post-harvest processing (MR: milled rice; BR: brown rice).

## References

[1] Gutteridge JM Halliwell B. Comments on review of free radicals in biology and medicine, by Barry Halliwell and John MC Gutteridge. *Free Radic Biol Med.* (1992) 12:93–5. 10.1016/0891-5849(92)90062-L 1537574

[2] Young IS Woodside JV. Antioxidants in health and disease. *J Clin Pathol.* (2001) 54:176–86. 10.1136/jcp.54.3.176 11253127 PMC1731363

[3] Willcox JK Ash SL Catignani GL. Antioxidants and prevention of chronic disease. *Crit Rev Food Sci Nutr.* (2004) 44:275–95. 10.1080/10408690490468489 15462130

[4] Halliwell B. Biochemistry of oxidative stress. *Biochem Soc Trans.* (2007) 35:1147–50. 10.1042/BST0351147 17956298

[5] Khush GS. What it will take to feed 5.0 billion rice consumers in 2030. *Plant Mol Biol.* (2005) 59:1–6. 10.1007/s11103-005-2159-5 16217597

[6] Sánchez B Rasmussen A Porter JR. Temperatures and the growth and development of maize and rice: a review. *Glob Chang Biol.* (2014) 20:408–17. 10.1111/gcb.12389 24038930

[7] Toriyama K Heong KL Hardy B. Rice is life: scientific perspectives for the 21st century. Proceedings of the World Rice Research Conference held in Tsukuba, Japan, 4-7 November 2004. InRice is life: scientific perspectives for the 21st century. *Proceedings of the World Rice Research Conference held in Tsukuba, Japan.* Los Banos: International Rice Research Institute (2005).

[8] Ha TY Ko SN Lee SM Kim HR Chung SH Kim SR Changes in nutraceutical lipid components of rice at different degrees of milling. *Eur J Lipid Sci Technol.* (2006) 108:175–81. 10.1002/ejlt.200500250

[9] Zhou Z Robards K Helliwell S Blanchard C. Composition and functional properties of rice. *Int J Food Sci.* (2002) 37:849–68. 10.1046/j.1365-2621.2002.00625.x

[10] Lerma-García MJ Herrero-Martínez JM Simó-Alfonso EF Mendonça CR Ramis-Ramos G. Composition, industrial processing and applications of rice bran γ-oryzanol. *Food Chem.* (2009) 115:389–404. 10.1016/j.foodchem.2009.01.063

[11] Miller A Engel KH. Content of γ-oryzanol and composition of steryl ferulates in brown rice (*Oryza sativa* L.) of European origin. *J Agric Food Chem.* (2006) 54:8127–33. 10.1021/jf061688n 17032019

[12] Goufo P Trindade H. Rice antioxidants: phenolic acids, flavonoids, anthocyanins, proanthocyanidins, tocopherols, tocotrienols, γ−oryzanol, and phytic acid. *Food Sci Nutr.* (2014) 2:75–104. 10.1002/fsn3.86 24804068 PMC3959956

[13] Akihisa T Yasukawa K Yamaura M Ukiya M Kimura Y Shimizu N Triterpene alcohol and sterol ferulates from rice bran and their anti-inflammatory effects. *J Agric Food Chem.* (2000) 48:2313–9. 10.1021/jf000135o 10888543

[14] Xu Z Hua N Godber JS. Antioxidant activity of tocopherols, tocotrienols, and γ-oryzanol components from rice bran against cholesterol oxidation accelerated by 2, 2’-azobis (2-methylpropionamidine) dihydrochloride. *J Agric Food Chem.* (2001) 49:2077–81. 10.1021/jf0012852 11308370

[15] Xu Z Godber JS. Antioxidant activities of major components of γ−oryzanol from rice bran using a linoleic acid model. *J Am Oil Chem Soc.* (2001) 78:645. 10.1007/s11746-001-0320-1

[16] Juliano C Cossu M Alamanni MC Piu L. Antioxidant activity of gamma-oryzanol: mechanism of action and its effect on oxidative stability of pharmaceutical oils. *Int J Pharm.* (2005) 299:146–54. 10.1016/j.ijpharm.2005.05.018 16005170

[17] Yasukawa K Akihisa T Kimura Y Tamura T Takido M. Inhibitory effect of cycloartenol ferulate, a component of rice bran, on tumor promotion in two-stage carcinogenesis in mouse skin. *Biol Pharm Bull.* (1998) 21:1072–6. 10.1248/bpb.21.1072 9821812

[18] Berger A Rein D Schäfer A Monnard I Gremaud G Lambelet P Similar cholesterol–lowering properties of rice bran oil, with varied γ–oryzanol, in mildly hypercholesterolemic men. *Eur J Nutr.* (2005) 44:163–73. 10.1007/s00394-004-0508-9 15309429

[19] Global Industry Analysts Inc. *Gamma Oryzanol – Global Market Trajectory & Analytics.* (2021). Available online at: https://www.researchandmarkets.com/reports/5302729/gamma-oryzanol-global-market-trajectory-and (Accessed June 15, 2022).

[20] Dong X Chen W Wang W Zhang H Liu X Luo J. Comprehensive profiling and natural variation of flavonoids in rice. *J Integr Plant Biol.* (2014) 56:876–86. 10.1111/jipb.12204 24730595

[21] Dong X Gao Y Chen W Wang W Gong L Liu X Spatiotemporal distribution of phenolamides and the genetics of natural variation of hydroxycinnamoyl spermidine in rice. *Mol Plant.* (2015) 8:111–21. 10.1016/j.molp.2014.11.003 25578276

[22] Kim NH Kwak J Baik JY Yoon MR Lee JS Yoon SW Changes in lipid substances in rice during grain development. *Phytochemistry.* (2015) 116:170–9. 10.1016/j.phytochem.2015.05.004 26021733

[23] Bollinedi H Singh N Krishnan SG Vinod KK Bhowmick PK Nagarajan M A novel LOX3-null allele (lox3-b) originated in the aromatic Basmati rice cultivars imparts storage stability to rice bran. *Food Chem.* (2022) 369:130887. 10.1016/j.foodchem.2021.130887 34461519

[24] Chen MH Bergman CJ. A rapid procedure for analysing rice bran tocopherol, tocotrienol and γ-oryzanol contents. *J Food Compos Anal.* (2005) 18:139–51. 10.1016/j.jfca.2003.09.004

[25] Tukey JW. *The Problem of Multiple Comparisons.* Princeton, NJ: Princeton University (1953).

[26] Saenchai C Prom-u-thai C Jamjod S Dell B Rerkasem B. Genotypic variation in milling depression of iron and zinc concentration in rice grain. *Plant Soil.* (2012) 361:271–8. 10.1007/s11104-012-1228-1

[27] Ram H Gandass N Sharma A Singh A Sonah H Deshmukh R Spatio-temporal distribution of micronutrients in rice grains and its regulation. *Crit Rev Biotechnol.* (2020) 40:490–507. 10.1080/07388551.2020.1742647 32204608

[28] Cho YH Lim SY Rehman A Farooq M Lee DJ. Characterization and quantification of γ-oryzanol in Korean rice landraces. *J Cereal Sci.* (2019) 88:150–6. 10.1016/j.jcs.2019.05.019

[29] Sun W Shi J Hong J Zhao G Wang W Zhang D Natural variation and underlying genetic loci of γ−oryzanol in Asian cultivated rice seeds. *Plant Genome.* (2022) 15:e20201. 10.1002/tpg2.20201 35762101 PMC12807011

[30] Barros J Escamilla-Trevino L Song L Rao X Serrani-Yarce JC Palacios MD 4-Coumarate 3-hydroxylase in the lignin biosynthesis pathway is a cytosolic ascorbate peroxidase. *Nat Commun.* (2019) 10:1994. 10.1038/s41467-019-10082-7 31040279 PMC6491607

[31] Lin PY Lai HM. Bioactive compounds in rice during grain development. *Food Chem.* (2011) 127:86–93. 10.1016/j.foodchem.2010.12.092

[32] Burden RS Cooke DT Carter GA. Inhibitors of sterol biosynthesis and growth in plants and fungi. *Phytochemistry.* (1989) 28:1791–804. 10.1016/S0031-9422(00)97862-2

[33] Laranjeira S Amorim-Silva V Esteban A Arró M Ferrer A Tavares RM Arabidopsis squalene epoxidase 3 (SQE3) complements SQE1 and is important for embryo development and bulk squalene epoxidase activity. *Mol plant.* (2015) 8:1090–102. 10.1016/j.molp.2015.02.007 25707755

[34] Shi J Gonzales RA Bhattacharyya MK. Identification and characterization of an S-adenosyl-L-methionine: Δ24-sterol-C-methyltransferase cDNA from soybean. *J Biol Chem.* (1996) 71:9384–9. 10.1074/jbc.271.16.9384 8621604

[35] Krishna AG Khatoon S Shiela PM Sarmandal CV Indira TN Mishra A. Effect of refining of crude rice bran oil on the retention of oryzanol in the refined oil. *J Am Oil Chem Soc.* (2001) 78:127–31. 10.1007/s11746-001-0232-0

[36] Bhaskaragoud G Rajath S Mahendra VP Kumar GS Krishna AG Kumar GS. Hypolipidemic mechanism of oryzanol components-ferulic acid and phytosterols. *Biochem Biophys Res Commun.* (2016) 476:82–9. 10.1016/j.bbrc.2016.05.053 27179780

[37] Huang SH Ng LT. Quantification of tocopherols, tocotrienols, and γ-oryzanol contents and their distribution in some commercial rice varieties in Taiwan. *J Agric Food Chem.* (2011) 59:11150–9. 10.1021/jf202884p 21942383

[38] Lamberts L De Bie E Vandeputte GE Veraverbeke WS Derycke V De Man W Effect of milling on colour and nutritional properties of rice. *Food Chem.* (2007) 100:1496–503. 10.1016/j.foodchem.2005.11.042

[39] Bollinedi H Yadav AK Vinod KK Gopala Krishnan S Bhowmick PK Nagarajan M Genome-wide association study reveals novel marker-trait associations (MTAs) governing the localization of Fe and Zn in the rice grain. *Front Genet.* (2020) 11:213. 10.3389/fgene.2020.00213 32391041 PMC7188789

[40] Gray PJ Conklin SD Todorov TI Kasko SM. Cooking rice in excess water reduces both arsenic and enriched vitamins in the cooked grain. *Food Addit Contam Part A Chem Anal Control Expo Risk Assess.* (2016) 33:78–85. 10.1080/19440049.2015.1103906 26515534

[41] Bollinedi H Dhakane-Lad J Krishnan SG Bhowmick PK Prabhu KV Singh NK Kinetics of β-carotene degradation under different storage conditions in transgenic Golden Rice§lines. *Food Chem.* (2019) 278:773–9. 10.1016/j.foodchem.2018.11.121 30583442

[42] Toyoizumi T Kosugi T Toyama Y Nakajima T. Effects of high-temperature cooking on the gamma-aminobutyric acid content and antioxidant capacity of germinated brown rice (*Oryza sativa* L.). *CYTA J Food.* (2021) 19:360–9. 10.1080/19476337.2021.1905721

[43] Pascual CD Massaretto IL Kawassaki F Barros RM Noldin JA Marquez UM. Effects of parboiling, storage and cooking on the levels of tocopherols, tocotrienols and γ-oryzanol in brown rice (*Oryza sativa* L.). *Food Res Int.* (2013) 50:676–81. 10.1016/j.foodres.2011.07.013

[44] Srichamnong W Thiyajai P Charoenkiatkul S. Conventional steaming retains tocols and γ-oryzanol better than boiling and frying in the jasmine rice variety Khao dok mali 105. *Food Chem.* (2016) 91:113–9. 10.1016/j.foodchem.2015.05.027 26258709

[45] Yuan GF Sun B Yuan J Wang QM. Effects of different cooking methods on health-promoting compounds of broccoli. *J Zhejiang Univ Sci B.* (2009) 10:580–8. 10.1631/jzus.B0920051 19650196 PMC2722699

[46] Zeng C. Effects of different cooking methods on the vitamin C content of selected vegetables. *Food Sci Nutr.* (2013) 43:438–43. 10.1108/NFS-11-2012-0123

[47] Silveira CM Moreira AV Martino HS Gomide RS Pinheiro SS Della Lucia CM Effect of cooking methods on the stability of thiamin and folic acid in fortified rice. *Int J Food Sci Nutr.* (2017) 68:179–87. 10.1080/09637486.2016.1226273 27592822

[48] Azam MM Padmavathi S Fiyaz RA Waris A Ramya KT Neeraja CN. Effect of different cooking methods on loss of iron and zinc micronutrients in fortified and non-fortified rice. *Saudi J Biol Sci.* (2021) 28:2886–94. 10.1016/j.sjbs.2021.02.021 34025166 PMC8117164

[49] Khuwijitjaru P Taengtieng N Changprasit S. Degradation of gamma-oryzanol in rice bran oil during heating: an analysis using derivative UV-spectrophotometry. *Silpakorn Univ Int J.* (2004) 4:154–65.

[50] Mishra R Sharma HK Sarkar BC Singh C. Thermal oxidation of rice bran oil during oven test and microwave heating. *J Food Sci Technol.* (2012) 49:221–7. 10.1007/s13197-011-0274-7 23572845 PMC3550867

[51] Thanonkaew A Wongyai S McClements DJ Decker EA. Effect of stabilization of rice bran by domestic heating on mechanical extraction yield, quality, and antioxidant properties of cold-pressed rice bran oil (*Oryza saltiva* L.). *LWT Food Sci Technol.* (2012) 48:231–6. 10.1016/j.lwt.2012.03.018

