# Supplementary figures and images for: From farm to plate: Spatio-temporal characterization revealed compositional changes and reduced retention of γ-oryzanol upon processing in rice
Source: Front Nutr. 2022 Nov 17;9:1040362. doi: 10.3389/fnut.2022.1040362 (PMC9712789; doi:10.3389/fnut.2022.1040362)

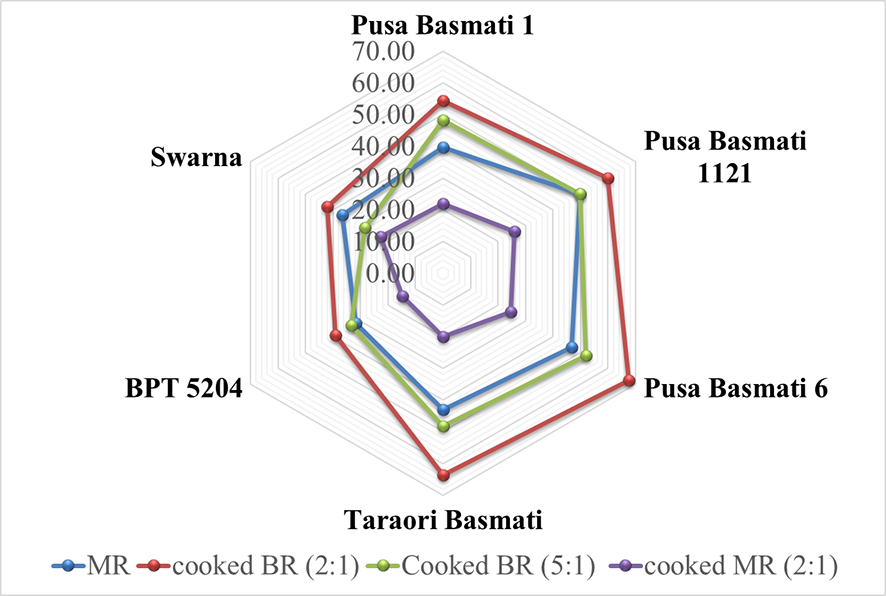

Supplement: Supplementary Figure 1 — Radar plot depicting the percentage retention of γ-oryzanol upon post-harvest processing (MR: milled rice; BR: brown rice). [file Image_1.TIF]
